# Supplementary material for: Optimized design of planar solid oxide fuel cell interconnectors
Source: PLoS One. 2024 Jul 3;19(7):e0298277. doi: 10.1371/journal.pone.0298277 (PMC11221743; doi:10.1371/journal.pone.0298277)
Supplement: Attachment — Submitted filename: Response to Reviewers.docx [file pone.0298277.s001.docx]

**Response letter**

**REVIEWER REPORTS**

**Reviewer Comments:**

**Reviewer 1**

The authors present a paper on the design of interconnects for planar Solid Oxide Fuel Cells geometry. Such topic is very important for the SOFC technology since the Interconnect is a critical component that has to ensure optimal gas distribution and electrical connection in order to prevent additional losses in efficiency of the system.

This work is providing 2D simulation of several parameters as the gas velocity, pressure, and hydrogen molar fraction distribution.

**Responses:** Many thanks to the reviewer for the above comments. According to the comments, we give the explanations and we have corrected the inaccurate information in the revised manuscript.

1. Authors compare the calculations performed on a “conventional” interconnect and a “novel” interconnect design, however the “conventional” design is just a simple geometry that is actually not in use for real systems. The “novel” design is something closer to the state-of-art interconnect design being characterized by the presence of channels with the function of distributing gases and contacts point aiming at collecting electrical currents. Even neglecting the feasibility of the proposed “novel” design, characterized by sharp edges and abrupt variations in geometry, which is not compatible with common manufacturing methods, I would expect a study on the electrical contribution too. The effect of geometry on the current collection should not be neglected. In fact, authors state this in the Section 1.3 but such aspect is not provided.

**Responses:** Thanks to the opinions of the referees. Based on several suggestions made by the reviewers, we have modified them.

2. Figure 3.1 is the same as Figure 2.3, I’m not sure is necessary to provide the same figure two times. It would be rather appropriate, for the sake of clarity, to add text boxes into the figures indicating the position and details of the sketch (for example:, rib, channel, electrode…). Also, the flow direction should be indicated by an arrow in the sketches, or at least an indication of the inlet and outlet positions.

**Responses:** The reviewer's suggestions are greatly appreciated. According to the reviewer's comments, we have made a correction to the picture.

3. In figures 4.1, 4.2, 4.3, to highlight the differences in the designs it would be helpful to indicate the quotes that are changing. It would be also helpful to indicate where the interconnect is in contact with the electrode and with the gas flow.

**Responses:** Thanks to the reviewer's suggestion, We have made modifications according to the reviewer's comments.

4. Several graphs lack the axis labels.

**Responses:** We have made modifications according to the reviewer's comments.

**Reviewer 2**

The findings and results in this study are meaningful.

The article provides an idea for a novel 2D simulation model for interconnector SOFCs, aiming to enhance

their performance. The findings and results in this study are meaningful. The introduction, results and conclusions of the manuscript are clear and well-written from the reviewer's perspective.

Although the authors needs point out more the differences between this article and previous research in the introduction, also the authors should reduce the similarity rate of the paper which is 37% in the current version.

**Response:** We are extremely grateful to the reviewer for the positive comments.
